# Spatial cluster analysis of human cases of Crimean Congo hemorrhagic fever reported in Pakistan

**DOI:** 10.1186/2049-9957-4-9

**Published:** 2015-03-02

**Authors:** Tariq Abbas, Muhammad Younus, Sayyad Aun Muhammad

**Affiliations:** Section of Veterinary Preventive Medicine and Public Health, University College of Veterinary and Animal Sciences, The Islamia University of Bahawalpur, Bahawalpur, Pakistan; Department of Pathobiology, College of Veterinary and Animal Sciences, Jhang, Pakistan; Department of Clinical Sciences, College of Veterinary and Animal Sciences, Jhang, Pakistan

**Keywords:** Crimean Congo hemorrhagic fever, Pakistan, Spatial autocorrelation, Clusters, Adjusted cumulative incidence

## Abstract

**Background:**

Crimean Congo hemorrhagic fever (CCHF) is a tick-borne viral zoonotic disease that has been reported in almost all geographic regions in Pakistan. The aim of this study was to identify spatial clusters of human cases of CCHF reported in country.

**Methods:**

Kulldorff’s spatial scan statisitc, Anselin’s Local Moran’s I and Getis Ord Gi* tests were applied on data (i.e. number of laboratory confirmed cases reported from each district during year 2013).

**Findings:**

The analyses revealed a large multi-district cluster of high CCHF incidence in the uplands of Balochistan province near it border with Afghanistan. The cluster comprised the following districts: Qilla Abdullah; Qilla Saifullah; Loralai, Quetta, Sibi, Chagai, and Mastung. Another cluster was detected in Punjab and included Rawalpindi district and a part of Islamabad.

**Conclusion:**

We provide empirical evidence of spatial clustering of human CCHF cases in the country. The districts in the clusters should be given priority in surveillance, control programs, and further research.

**Electronic supplementary material:**

The online version of this article (doi:10.1186/2049-9957-4-9) contains supplementary material, which is available to authorized users.

## Multilingual abstracts

Please see Additional file 1 for translations of the abstract into the six official working languages of the United Nations.

## Introduction

Crimean-Congo hemorrhagic fever (CCHF) is an arboviral zoonotic infection with potential for human-to-human transmission. The disease is widely distributed across Africa, Asia, and Europe. It is generally asymptomatic in infected animals (usually domestic livestock), but highly fatal in humans with a 10 to 50% case fatality [1]. The infection is transmitted to humans by infected ticks, direct contact with meat or blood of infected animals, or direct contact with the blood or secretions of an infected person [2]. The causative virus of CCHF is a member of the genus *Nairovirus* in the family *Bunyaviridae.* It is an enveloped virus containing negative-sense; single-stranded RNA composed of three segments. The virus has been classified into seven genotypes (Asia-1, Asia-2, Africa-1, Africa-2, Africa-3, Euro-1, and Euro-2) that coincide well with their geographical regions [3].

The first confirmed case of CCHF in Pakistan was reported in 1976 followed by outbreaks from almost all areas of the country mainly Balochistan province where the disease still remains entrenched. Genetic analysis of the viruses has confirmed the presence of Asia-1 and Asia-2 genotypes in this province. In Pakistan, CCHF exhibits two annual peaks from March to October [4]. Since 2000, there is an increasing trend in the number of cases, for instance during 2012–2013, a total of 162 cases including 38 deaths reported in different areas [5]. The disease incidence continues to expand and cover previously uninfected areas across all four provinces. Locating areas with relatively high incidence is essential in order to target surveillance and prevention programmes. The purpose of this study was to explore data about human CCHF cases for presence of spatial clusters of the disease in Pakistan.

## Methods

Information about the occurrence of the disease was extracted from the Weekly Epidemiological Bulletin(s) jointly published by the National Institute of Health (NIH), Islamabad and the country office of the World Health Organization (WHO), Pakistan. The dataset included total count of laboratory confirmed human cases of CCHF reported from different country areas during January to December, 2013. SaTScan™ v9.3.1 and GeoDa™ v.1.6.6 software were used to execute the analyses. Population of each district was extrapolated from the linear growth rate between the censuses of 1981 and 1998. Incidence per million of population was calculated for each district, with the total number of cases as the event variable and the estimated population for 2013 as the base variable. The spatial empirical Bayes (EB) smoothing method was applied to adjust incidence proportion estimates using first order queen contiguity weights. The spatial EB method adjusts rates toward the average of the neighbors rather than the overall mean of the study area. There were no reliable population data for northern areas and disputed Kashmir; therefore these regions were excluded from the analyses.

Global Moran’s *I* statistic was used to explore data for evidence of spatial clustering at the country level. The results of the test vary between + 1.0 and -1.0. The Moran’s *I* > 0, = 0, and < 0 indicate the positive spatial autocorrelation, random distribution, and negative spatial autocorrelation, respectively. In order to identify the location of the clusters, spatial scan statistic and local indicators of spatial autocorrelation, local Moran’s *I* and Getis ord Gi* were also used. The two techniques have been used simultaneously to complement the findings. The number of the permutation test was set to 999 and the significance level was set to 0.05. The formulas of the techniques are given in the Appendix.

Kulldorff’s spatial scan statistic has been one of the most widely used statistical methods for exploring spatial clusters. The spatial scan statistic has been utilized for a variety of diseases including vector-borne pathogens such as CCHF [6]. It uses moving windows of varying diameter to evaluate clusters. The radii of those windows range from zero to an upper limit set by the user. For each window location and size, the software compares a value inside the window to the value outside the window. This, in turn, calculates the likelihood function for each window. Under the Poisson assumption, the likelihood function for a specific window is proportional to:


where *C* is the total number of cases, c is the observed number of cases within the window, *E* [*c*] is the expected number of cases within the window under the null hypothesis (assuming constant risk in the study domain), and *I*() is the indicator function which is equal to 1 when the window is more than expected [7]. A retrospective purely spatial scan statistic was applied to detect high -risk clusters of CCHF with a discrete Poisson model. The maximum spatial cluster size of 50% of the population at risk was applied to avoid pre-selection bias.

## Results

**Figure 1:**
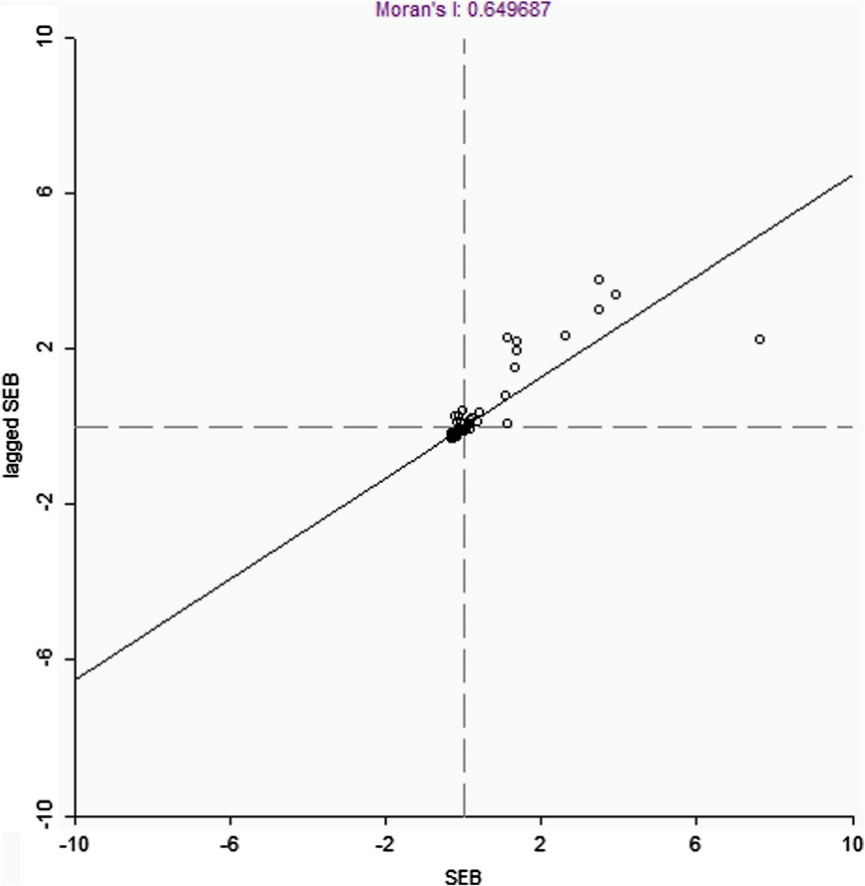
**Moran scatter plot of adjusted cumulative incidence (CI) of reported CCHF case in various districts.** The horizontal axis shows the standardized value of CCHF adjusted cumulative incidence (CI), the vertical axis shows the spatial average of the standardized value. The slope of the regression corresponds to the global Moran’s I value which is 0.649687.

A total of sixty-seven laboratory confirmed cases were reported from thirty-one districts. The adjusted incidence in various districts ranged from 0 to 19 cases per million. Global Moran’s *I* statistic was used to explore data for evidence of spatial clustering at the country level. The result showed positive spatial autocorrelation in adjusted incidence (Moran’s *I* = 0.6597, P < 0.05). Figure 1 displays the Moran scatter plot of adjusted incidence of reported CCHF cases in various districts. Data are standardized so that units on the graph are expressed in standard deviations from the mean. The horizontal axis shows the standardized value of “CCHF incidence” for a district, the vertical axis shows the standardized value (spatial lag) of the average incidence for that district’s neighbors as defined by the weights matrix. The slope of the regression expresses the global Moran’s I value which is 0.649687. The graph is divided into four quadrants: high-high (upper right) and low-low (lower left), which both denote positive spatial autocorrelation, high-low (lower right) and low- high (upper left), which both donate negative spatial autocorrelation.

**Figure 2:**
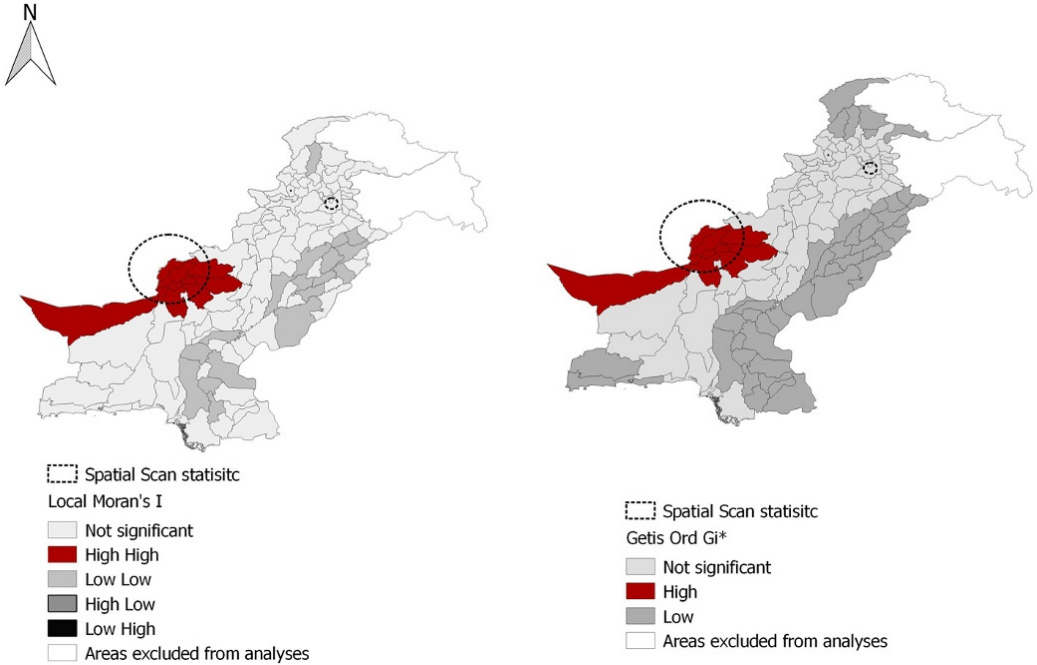
Map of Pakistan displaying output of spatial scan statistic, Local Moran’s I and Getis- Ord Gi* tests. Local Moran’s I identified high - high (red) and low-low (medium gray) clusters. However, not even a single high-low (dark gray) or low-high (black) type spatial outlier could be found. Getis- Ord Gi* detected hot spots (red) and cold spots (gray) of the disease cases across the country. The map also illustrates location of two most likely cluster clusters detected through spatial scan statistic (doted circles).

Spatial scan statistic unmasked two most likely clusters in the country (see Figure 2). A large cluster was detected in the Balochistan near its border with the Afghanistan. The cluster included the following districts: Qilla Abdullah; Qilla Saifullah; Loralai, Quetta, Sibi; Chagai and Mastung. Another cluster was found in Punjab. It included the Rawalpindi district and a part of Islamabad. Getis Ord Gi* statistic classified 9 districts as hotspots, 45 districts as cold spots where as 69 districts were found as non-significant. Local Moran’s *I* identified 08 districts as high-high clusters, 18 districts as low-low clusters where as 96 districts were classified as non-significant. Local Moran’s *I* could not found any type of spatial outlier. Mathematically, these techniques differ from each other; this has lead to apparent differences in final output map.

## Discussion

Studies conducted in Iran and Turkey have also found spatial clustering of CCHF [6, 8]. Khushal *et al.*[9] studied the residential location of CCHF cases admitted to three public sector hospitals of Quetta –the capital city of Balochistan. The districts with a relatively high prevalence were located in cluster detected in the province. Alam et al. [3] published a map on the distribution of IgM confirmed CCHF cases reported to National Institute of Health, Islamabad (2003 to 2008). All affected districts were located in clusters identified in our study.

The reasons for detection of disease cluster in Balochistan are unknown. The disease is endemic in this province since 1987. Many cases from Afghanistan are referred to tertiary care hospitals in Quetta city [3]. The province lies on migration routes of the nomads [10]. Moreover, there is cross-border trade of animal’s skins and hides through Balochistan [11]. More than 60% of all human cases of CCHF in Iran have been reported in Sistan and Baluchistan Province. This has been attributed to excessive livestock import and high frequency of Afghani traveling to these areas [12]. In Pakistan, livestock trade and movement during eid ul azha has potential to spread infection in the country. The animals are brought for sale in cities such as Rawalpindi [13]. Relationship between altitude and CCHF occurrence has been investigated. The altitude has been supposed to affect tick distribution and animal husbandry practices [14]. Estrada-Peña *et al.*[6] modeled the spatial distribution of crimean-congo hemorrhagic fever outbreaks in Turkey and found that areas with higher risk of CCHF were correlated with the zones of high climate suitability for the tick together with a high rate of fragmentation of agricultural land interspersed between forest and shrub-type vegetation. Yagci-Caglayik *et al.*[15] studied seroprevalence and risk factors of Crimean-Congo hemorrhagic fever in selected seven provinces of Turkey. The most important risk factors for CCHF seropositivity were older age, male gender, illiterate, farmer, animal husbandry, living in rural residence in adobe houses, and a previous tick bite history.

It is important to note that each of the techniques used in this study has its own advantages and limitations. The spatial scan statistic has several advantages over other clustering detection methods because it: i) accounts for problem of multiple testing, ii) adjusts for the heterogeneous population and any number of categorical covariates, and iii) searches for clusters without specifying their size or location thereby overcoming the problem of pre-selection bias. However, SaTScan, often reports heterogeneous clusters i.e. a large cluster may contain a number of low- risk areas. Moreover, it is sensitive to user-defined settings [16]. The shapes of clusters may not always be circular or elliptical. The Getis Ord Gi* identifies clusters of high or low values but cannot capture negative spatial autocorrelation (spatial outliers). Local Moran’s *I* statistic, on the other hand, has the ability to detect both positive and negative spatial correlations. The Gi* statistic has been preferred in some studies as it matches the usual definition of cluster and is indicated for use with variables that posse natural origin [17]. Geary’s *C* is another measure of spatial autocorrelation. It emphasizes on differences in values between pairs of observations. sensitive to differences in small neighborhoods and statistically less well behaved compared to local Moran’s *I*. Therefore Moran’s I is usually preferred to Geary’s *C*[18].

Limitations of this study included the use of secondary data and the problems inherent to data quality. Spatial health data might have uncertainty in location (i.e. place of residence and location where the attributes recorded were not same), underreporting and mis-diagnosis. The level of those uncertainties in disease data is unknown and cannot be controlled. The disease patterns are scale dependent and subjected to temporal changes. These facts should be considered in interpretation of the findings. We extrapolated population from inter census growth rate from 1981 to 1998. Population count data are generally stable so it was quite acceptable to extrapolate.

## Conclusion

In short, CCHF is an emerging zoonotic disease in Pakistan. Understanding the spatial patterns of the disease is important in order to make rational use of the limited resources. Our study provides empirical evidence for the existence of statistically significant spatial clusters of high CCHF incidence in few districts of Balochistan, Rawalpindi and Islamabad. Those districts may be targeted for research and healthcare interventions on priority such as public awareness programmes, one health initiative etc. Our findings emphasize to initiate sustained and comprehensive surveillance activities focusing timely detection and disease control strategies in these areas. The location of a cluster close to Afghanistan border highlights the importance of the findings for organizations dealing with the disease at the national, regional and global levels.

## Appendix



where *n* is total number of distircts, *W*_*ij*_ is the meaure of spatial proximity between *i* and *j* districts, *y*_*i*_ and *y*_*j*_ is the incidence in distirict *i* and adjacent district *j*, and  is the mean incidence of all districts in the country. The numerator is obtained by multiplying the number of districts by the product of deviation from the mean for all pairs of adjacent disticts, the denominator is the product of the sum of the spatial weights, and the square of deviation for the mean for all pairs of adjacent disticts.


 where *x*_*i*_ is the incidence in distric,  is the mean of incidence from all districts, *SD*_*x*_ is the standard deviation of incidence from all districts, and *W*_*ij*_ is the row standardized spatial weights.


where *x* represents CCHF incidence within a given district, *W*_*ij*_ is a spatial weight which defines neighboring districts *j* to *I*, *W*_*i*_ is the sum of weights *W*_*ij*_,  is the mean of the country level CCHF incidence, , *s* is the standard deviation of the *x* values.

## Electronic supplementary material

Additional file 1: **Multilingual abstracts in the six official working languages of the United Nations.** (PDF 313 KB)
