# Supplementary material for: Spatial cluster analysis of human cases of Crimean Congo hemorrhagic fever reported in Pakistan
Source: Infect Dis Poverty. 2015 Mar 2;4:9. doi: 10.1186/2049-9957-4-9 (PMC4429983; doi:10.1186/2049-9957-4-9)
Supplement: Supplementary file 1 — Additional file 1: Multilingual abstracts in the six official working languages of the United Nations. (PDF 313 KB) [file 40249_2014_99_MOESM1_ESM.pdf]

Translation of the abstract into the six official working languages of the United Nations

تحليل التجمع الحيّزي للحالات البشرية من حمى الكونغو والقرم النزفية المبلغ عنها في الباكستان

طارق عباس، يونس محمد، سيد عون محمد

الملخّص

الخلفية: حمى الكونغو والقرم النزفية **(CCHF)** هي مرض فيروسي حيواني المنشأ منقول بالقراد ذكر حدوثه في كل مناطق الباكستان تقريبا. تهدف هذه الدراسة إلى تحديد التوزع الحيّزي لحالات **CCHF** البشرية المبلغ عن حدوثها في البلاد.

الطرق: جرى تطبيق كل من احصائيات المسح الحيّزي لكلدورف، اختبارات **I** المحلية (Anselin's Local Moran's) و(Getis Ord Gi*) على البيانات (أي عدد الحالات المثبتة مخبريا والمبلغ عن حدوثها من كل مقاطعة خلال عام 2013).

النتائج: كشفت تحليل البيانات عن حدوث عدد مرتفع من حالات حمى الكونغو والقرم النزفية بتجمعات كبيرة متعددة المناطق في المناطق المرتفعة في مقاطعة بلوشستان قرب الحدود الأفغانية. وقد ضمت التجمعات المقاطعات التالية: قلة عبدالله، قلة سيف الله، لورالاي، كويتا، سيبي، شاغاي وماستونغ. وقد أمكن العثور على تجمع آخر في البنجاب وقد ضم مقاطعة روالبندي وقسما من إسلام أباد.

الاستنتاج: إننا نقدم دليلا تجريبيا على التجمع الحيّزي لحالات حمى الكونغو والقرم النزفية في البلاد. يجب إيلاء المقاطعات الموجودة في التجمعات أولوية لدى استقصاء حالات المرض ، ووضع برامج السيطرة عليها، وإجراء الأبحاث الوبائية الخاصة بـها .

Translated from English version into Arabic by Lina SM, through

TRANSLATORS
WITHOUT BORDERS

巴基斯坦人感染克里米亚-刚果出血热病例的空间聚集分析

Tariq Abbas, Younus Muhammad, Sayyad Aun Muhammad

摘要

引言：克里米亚-刚果出血热(CCHF)是经蜱传播的病毒性的动物源性疾病，几乎在巴基斯坦境内的所有地区均有病例报告。本研究的目的是确定巴基斯坦该病的空间分布情况。

方法：采用 Kulldorff's 空间扫描统计、聚类和异常值分析(Anselin Local Moran's I)和热点分析(Getis-Ord Gi*)方法对 2013 年各地区报告的实验室确诊病例进行分析。

结果：数据分析结果显示，在内陆俾路支省与阿富汗交界的边境地区呈现跨高发病率、多地区的聚集区。该集群包括以下地区，即 Qilla Abdullah、Qilla Saifullah、Loralai、Quetta、Sibi、Chagai 和 Mastung。另一个集群检测了旁遮普（Punajb）地区的情况，包括包括拉瓦尔品第区（Rawalpindi）和伊斯兰堡的一部分。

结论：该研究为巴基斯坦人 CCHF 病例的空间聚集性提供了资料。在上述地区需要加强对该病的监测，疾病控制项目支持和开展相应的流行病学调查研究。

Translated from English version into Chinese by Zhang Shao-sen, through

CHINESE CENTER FOR DISEASE CONTROL AND PREVENTION
NATIONAL INSTITUTE OF PARASITIC DISEASES

Analyse typologique spatiale des cas humains de fièvre hémorragique de Crimée-Congo signalés au Pakistan

Tariq Abbas, Younus Muhammad, Sayyad Aun Muhmmad

Résumé

Contexte : La fièvre hémorragique de Crimée-Congo (FHCC) est une zoonose virale transmise par les tiques qui a été signalée dans presque toutes les régions du Pakistan. Le but de la présente étude était d'identifier la typologie spatiale des cas humains de FHCC signalés dans le pays.

Méthodes : La statistique de scan spatiale de Kulldorff, l'analyse de l'Indice de Moran (Anselin) et les tests Getis-Ord Gi* ont été appliqués aux données (c.-à-d. le nombre de cas signalés dans chaque district pendant l'année 2013 et confirmés en laboratoire).

Résultats : Les analyses ont mis en évidence un vaste groupe multi-district de forte incidence de la FHCC sur les hautes terres de la province du Baloutchistan près de la frontière afghane. Ce groupe comprend les districts suivants : Qilla Abdullah, Qilla Saifullah, Loralai, Quetta, Sibi, Chagai et Mastung. Un autre groupe a été détecté dans le Pendjab et comprend le district de Rawalpindi ainsi qu'une partie d'Islamabad.

Conclusion : Nous apportons des preuves empiriques du regroupement spatial des cas humains de FHCC dans le pays. Les districts compris dans ces groupes doivent être prioritaires pour la surveillance, les programmes de contrôle, et les nouvelles recherches.

Translated from English version into French by Nathalie Hecker, through

TRANSLATORS WITHOUT BORDERS

Территориально-групповой анализ случаев заболевания людей геморрагической лихорадкой Крым-Конго в Пакистане

Тарик Аббас, Юнус Мухаммед, Сайад Аун Мухмад

Краткое изложение

История вопроса: Геморрагическая лихорадка Крым-Конго – это вирусное зоонозное заболевание, передающееся через укусы клещей, которое было зарегистрировано в почти всех географических регионах Пакистана. Цель данного исследования – определить территориальные группы случаев заболевания людей геморрагической лихорадкой Крым-Конго, зарегистрированных в стране.

Методы: Статистический метод группированных выборок Куллдорфа; на данных были проведены тесты Anselin Local Moran I и Getis Ord Gi* (т.е. количество подтвержденных случаев заболевания в каждом районе в течение 2013 г.).

Результаты: Анализы выявили большую, включающую несколько районов, группу с высокой частотой геморрагической лихорадки Крым-Конго в нагорьях провинции Балочистан, рядом с границей с Афганистаном. Группа включает следующие районы: Кила-Абдулла, Килла-Сайфулла, Лоралай, Кветта,

Сиби, Чагай и Мастунг. Другая группа была обнаружена в Пенджабе и включает район Равалпинди и часть Исламабада.

Заключение: Мы предоставляем эмпирические доказательства наличия территориальных групп случаев заболевания людей геморрагической лихорадкой Крым-Конго. Районам, входящим в эти территориальные группы, должно уделяться первоочередное внимание при наблюдении, внедрении программ контроля и дальнейших исследованиях.

Translated from English version into Russian by Elena McDonnell, through

Análisis de conglomerados espaciales de casos de fiebre hemorrágica de Crimea-Congo en seres humanos declarados en Pakistán

Tariq Abbas, Younus Muhammad, Sayyad Aun Muhmmad

Resumen

Antecedentes: La fiebre hemorrágica de Crimea-Congo (FHCC) es una zoonosis vírica transmitida por garrapatas y declarada en casi todas las regiones geográficas de Pakistán. El objetivo del presente estudio ha sido la detección de los conglomerados espaciales de casos de FHCC en seres humanos declarados en el país.

Métodos: En los datos se han aplicado las técnicas de estadística espacial de Kulldorff, el indicador I de Moran de Anselin y el Gi de Getis y Ord (es decir, número de casos confirmados clínicamente declarados desde cada distrito durante el año 2013).

Resultados: Los análisis mostraron un gran conglomerado de varios distritos con una gran incidencia de FHCC en las montañas de Beluchistán, cerca de la frontera con Afganistán. El conglomerado abarcaba los siguientes distritos:Qilla Abdullah; Qilla Saifullah; Loralai, Quetta, Sibi, Chagai y Mastung. Se ha detectado otro conglomerado en Punjab que incluye el distrito de Rawalpindi y parte de Islamabad.

Conclusión: Aportamos pruebas empíricas de conglomerados espaciales de casos de FHCC en humanos en el país. Se debe dar prioridad a los distritos comprendidos en los conglomerados en cuanto a supervisión, programas de control e investigaciones posteriores.

Translated from English version into Spanish by Raquel Bentué, through
